# The economic burden of individuals living with generalized myasthenia gravis and facing social determinants of health challenges

**DOI:** 10.3389/fpubh.2023.1247931

**Published:** 2023-09-12

**Authors:** Tom Hughes, James F. Howard, Nicholas J. Silvestri, Ashley E. L. Anderson, Mai Sato, Sharon Suchotliff, Jeffrey T. Guptill, Glenn Phillips

**Affiliations:** ^1^Argenx US Inc., Boston, MA, United States; ^2^Department of Neurology, University of North Carolina, Chapel Hill, NC, United States; ^3^Department of Neurology, University at Buffalo, Buffalo, NY, United States; ^4^Department of Neurology, Houston Methodist, Houston, TX, United States; ^5^ZS Associates, New York, NY, United States

**Keywords:** myasthenia gravis, social determinants of health, economic burden, mixed methods, patient support

## Abstract

**Objective:**

Better understanding the impact of social determinants of health (SDOH) barriers from the patient perspective is crucial to improve holistic patient support in generalized myasthenia gravis (gMG), a rare autoimmune disorder with high disease and treatment burden. The objective of this study was to identify economic challenges experienced by individuals living with gMG and SDOH barriers to better address current unmet needs.

**Methods:**

Adults (18–75 years) living with gMG and experiencing SDOH barriers in the United States were recruited to a mixed-methods study including qualitative interviews and a web-based quantitative survey. Quotas were implemented to include a balanced spread of baseline demographic categories including insurance type, living environment, and employment status among the study sample. Direct and indirect economic challenges were identified by degree of concern.

**Results:**

The survey was completed by 38 individuals living with gMG, the majority of whom were enrolled in public insurance and not employed. The most commonly reported major economic concerns were managing funds for emergency care (66%), loss of income (61%), and non-medical expenses (58%), highlighting the diversity of economic challenges. Individuals who were using public insurance plans, living in non-urban environments, and unemployed experienced pronounced challenges around managing non-medical costs and accessing government assistance.

**Conclusion:**

Both direct and indirect costs were emphasized as major concerns among individuals living with gMG and SDOH barriers. Increasing access to relevant, personalized, and holistic resources, including care management, should be prioritized to improve disease management and outcomes for individuals living with gMG.

## 1. Introduction

Generalized myasthenia gravis (gMG), a rare autoimmune disorder associated with the failure of neuromuscular junction transmission, currently affects more than 60,000 individuals in the United States (US) (1–5). Fluctuating and debilitating weakness in ocular, facial, bulbar, axial, and limb muscles is a hallmark of gMG and contributes to substantial patient burden despite available treatment options (6–10).

In addition to clinical and humanistic burden, high economic burden has been reported in gMG globally across Europe (11–15), Asia (16–20), Australia (21), South America (22), and North America (14, 15, 23–27), with the most robust evidence for direct medical costs (28). In the US, where direct medical costs are particularly high, the cost of gMG inpatient care rose 13-fold between 2003 and 2013 (23), which can be attributed in part to the steadily rising prevalence of gMG (ranging from 150 to 200 cases per million) due to improvements in disease recognition and diagnosis (4). However, practice pattern changes in gMG care are likely a larger influence, with an over 6-fold increase in hospital discharges observed between 2003 and 2013 (23). Moreover, the proportion of discharges with use of intravenous immunoglobulin increased from 10% in 2000 to 28% in 2005, driving costs as some public and private insurers can be restrictive of outpatient infusion of intravenous immunoglobulin (23). As the treatment landscape continues to evolve with new therapeutics offering hope for improved outcomes (2, 29–31), it is critical to continue pursuing innovative access solutions so that cost is not a limiting factor for delivering treatments to those who can benefit.

Individuals living with gMG are also highly impacted by other types of costs contributing to their economic burden, though such evidence is much more limited. These include indirect costs (expenses incurred due to loss of or reduction in employment or productivity for both affected individuals and their caregivers) reported across Bulgaria (11), Denmark (32), Germany (12), India (16), Australia (21, 33), and the US (11, 12, 16, 21, 28, 34), as well as direct non-medical costs (direct expenses related to accessing healthcare and supporting other aspects of living with the illness) reported in Australia (21), Bulgaria (11), India (16), and Germany (12). As the impact of gMG on activities of daily living can be severely disabling (21), it is not surprising that lifestyle changes necessitated by a gMG diagnosis can accumulate additional cost burden across many different dimensions of daily life.

Growing evidence substantiates that social determinants of health (SDOH)—including the conditions in which people are born, grow, work, live, and age—affect health, functioning, and health-related quality of life outcomes (35, 36). Importantly, SDOH barriers exacerbate economic burden, or “financial toxicity,” which is one of the core issues underlying health inequities historically (37, 38). Such economic impact of SDOH has been demonstrated among more common chronic conditions and some rare diseases (38–40), but the literature is still scarce in gMG. While a Bulgarian study reported that employment status significantly impacted the total cost of gMG (11), additional robust evidence, especially based in the US, is currently outdated or lacking. This critical knowledge gap stems from the historical underrepresentation of individuals living with SDOH barriers in research, in part because conventional recruiting approaches often do not systematically reach or resonate with individuals experiencing complex SDOH challenges (41).

To address unmet needs related to the economic burden of gMG, it is essential to better understand the economic burden of SDOH in gMG from the patient’s perspective. The objectives of this study were to identify common economic challenges faced by individuals living with gMG and SDOH barriers and to determine which SDOH factors can be potential drivers. With the insights gained from the study, we discuss how to better support the gMG community using an increasingly holistic approach.

## 2. Materials and methods

### 2.1. Study design and ethics

This mixed-methods study included qualitative interviews and a cross-sectional multimodal quantitative survey. The full methodology has been previously published (42). All participants received compensation for their participation in this study, and protocols and materials used in the study received Institutional Review Board approval (IRB#20220823, WCG IRB, Puyallup, WA, USA).

### 2.2. Participant recruitment and inclusion criteria

To overcome the inherent challenges of recruiting a diverse cohort of individuals living with gMG in the US, a combination of recruiting strategies was used for both the qualitative and quantitative phases of the study. In addition to partnering with multiple third-party vendors with robust rare disease networks, recruiting strategies also included close collaboration with gMG patient advocacy groups (PAGs; MG Holistic Society and MG Georgia) to extend the reach of recruitment to individuals with gMG who may not be registered in conventional research networks. PAG collaborators were supplied with a simple and illustrative “one-pager” flyer describing the purpose, inclusion criteria, and information about the research, which were shared across wider peer groups of individuals living with gMG (including those residing on social media platforms) to engage potential participants.

To ensure a diverse sample, a screener questionnaire was utilized to implement quotas based on pre-defined SDOH factors including age, gender, racial or ethnic background, employment status, living environment, insurance status, and level of education (Supplementary Table 1). For the qualitative interview phase only, caregivers and PAG representatives with experience caring for or working with individuals in the US living with gMG and SDOH barriers were also recruited using separate inclusion criteria (Supplementary Table 2). Many individuals living with gMG require care and support in their daily living, and PAGs play a robust role in fostering and supporting communities for diagnosed individuals. By incorporating their critical input, we aimed to capture a well-rounded perspective of the impact of SDOH on the experience of individuals living with gMG to inform the survey design and hypotheses.

### 2.3. Qualitative interviews

Double-blinded, 45-min, web-assisted phone interviews were moderated by an academic researcher with expertise in engaging and facilitating conversations around SDOH. In accordance with a discussion guide, interviewees were asked questions regarding their experiences and challenges faced at diagnosis, in treatment decision-making, post-diagnosis, and in accessing support (Supplementary Table 3). Descriptive themes and insights collected from the qualitative interviews were utilized to inform the quantitative survey design.

### 2.4. Quantitative survey

Survey design was informed by learnings and concepts gathered from the qualitative interviews. The web-based survey consisted of multiple-choice questions spanning a wide range of experiences and perceptions of living with gMG and was designed to be completed in approximately 20 min (Supplementary Table 4). Regarding economic burden, a list of 15 potential concerns related to costs, economic challenges, and accessing care that was compiled based on published literature and interview insights was presented. A subset of these potential concerns included those alluding to direct medical costs (managing funds for emergency care, cost of medications and treatments, cost of medical care), indirect costs (loss of income), and direct non-medical costs (cost of non-medical expenses, cost of accessible transportation, cost of home modifications, cost of professional caregiving, cost of childcare). Respondents were asked to select their degree of concern (major concern, concern, or no concern) for each statement, which were shown in randomized order to minimize bias. The survey and optional telephone assistance were offered in English or Spanish, and caregivers were allowed to assist individuals in completing the survey. De-identified data were aggregated and analyzed for the overall cohort and by SDOH subgroups based on screener data.

## 3. Results

### 3.1. Participant demographics and characteristics

A total of 15 individuals participated in the qualitative interviews, of which 11 were individuals living with gMG and SDOH challenges, 2 were caregivers of such individuals, and 2 were gMG PAG representatives experienced in working with diverse gMG communities. In the quantitative phase, a total of 38 individuals living with gMG and SDOH challenges completed the survey. Across both phases, diverse cohorts of individuals living with gMG between the ages of 18 and 75 years across all pre-defined SDOH factors were recruited, with a high proportion using Medicaid, using Medicare, and/or unemployed (Table 1).

**Table 1:** Baseline demographics and characteristics of participants living with gMG.

	Interviews, *n* (*N* = 11^*^)	Survey, *n* (*N* = 38)
Age		
18–40 years	3	9
41–60 years	7	20
61–75 years	1	9
Gender		
Men	4	9
Women	7	28
Binary gender nonconforming	0	1
Current insurance type^†^		
Medicaid	5	13
Medicare	3	17
Private insurance	1	7
Other	3	1
Living environment		
Rural/small town	4	12
Suburban	3	13
Urban	4	13
Employment status		
Not employed	9	23
Retired	0	5
Employed (including self-employed)	2	10
Level of education		
GED/high school	2	11
Post-secondary	9	23
Prefer not to answer^‡^	0	4
Race/ethnic background		
Non-White/Caucasian^§^	8	23
White/Caucasian	3	15

GED, General Education Development; gMG, generalized myasthenia gravis.

* Of the 15 total participants in the interviews, baseline demographics and characteristics are only shown for individuals living with gMG (*n* = 11). The remaining 4 participants in the interviews were caregivers (*n* = 2) and PAG representatives (*n* = 2) who fulfilled the inclusion criteria outlined in Supplementary Table 2.

† Insurance type totals may not add up to the total sample size as respondents could choose multiple options if applicable. “Private” insurance included commercial and employer-provided insurance. “Other” insurance included Veterans Affairs and self-purchased insurance. Individuals who responded with “Other” insurance were excluded from the insurance type–based subgroup analyses.

‡ Participants who preferred not to answer were excluded from this subgroup analysis.

§ Non-White/Caucasian included Hispanic/Latin@, Black/African American, Native American/Indigenous Person, Asian/Pacific Islander, and Middle Eastern or North African.

### 3.2. Qualitative research results

Throughout the 15 qualitative interviews conducted with 11 individuals living with gMG and SDOH barriers, 2 caregivers of such individuals, and 2 gMG PAG representatives, compounding economic burden was highlighted for individuals using public insurance, living in non-urban environments, or unemployed. Individuals using Medicaid expressed that their insurance did not always cover certain treatments their physicians recommended and that paying out of pocket for uncovered expenses was challenging. One unemployed individual stated, “*I could not work because of my gMG. Trying [to work] was taking a toll on me mentally, and eventually I lost my job that I loved and over $40,000 of income.”*

More specifically, challenges in paying for non-medical expenses, including accessible transportation and professional caregiving, were expressed. An individual living in a non-urban environment stated, *“The cost of gas going back and forth to go into another town to get [treatment]. It’s a long day and you are exhausted; you need to travel and spend money on getting something to eat… it’s ridiculous.”* A caregiver for an individual living with gMG using Medicare stated, *“It’s difficult to take Mom on appointments and focus on my work,”* illustrating additional caregiver burden.

Additionally, challenges in applying for government assistance were acutely demonstrated by an unemployed individual using Medicare who stated, *“I was waiting for disability approval, which took 3 years in economic limbo.”* A PAG representative also reinforced access challenges for those living with SDOH barriers, stating, *“We try to assist these [individuals] in finding funding and bringing them closer to the assistance programs.”* These insights collected from the interviews were utilized to inform the survey design for the quantitative phase.

### 3.3. Common types of economic concerns among individuals living with gMG and SDOH barriers

**Figure 1:**
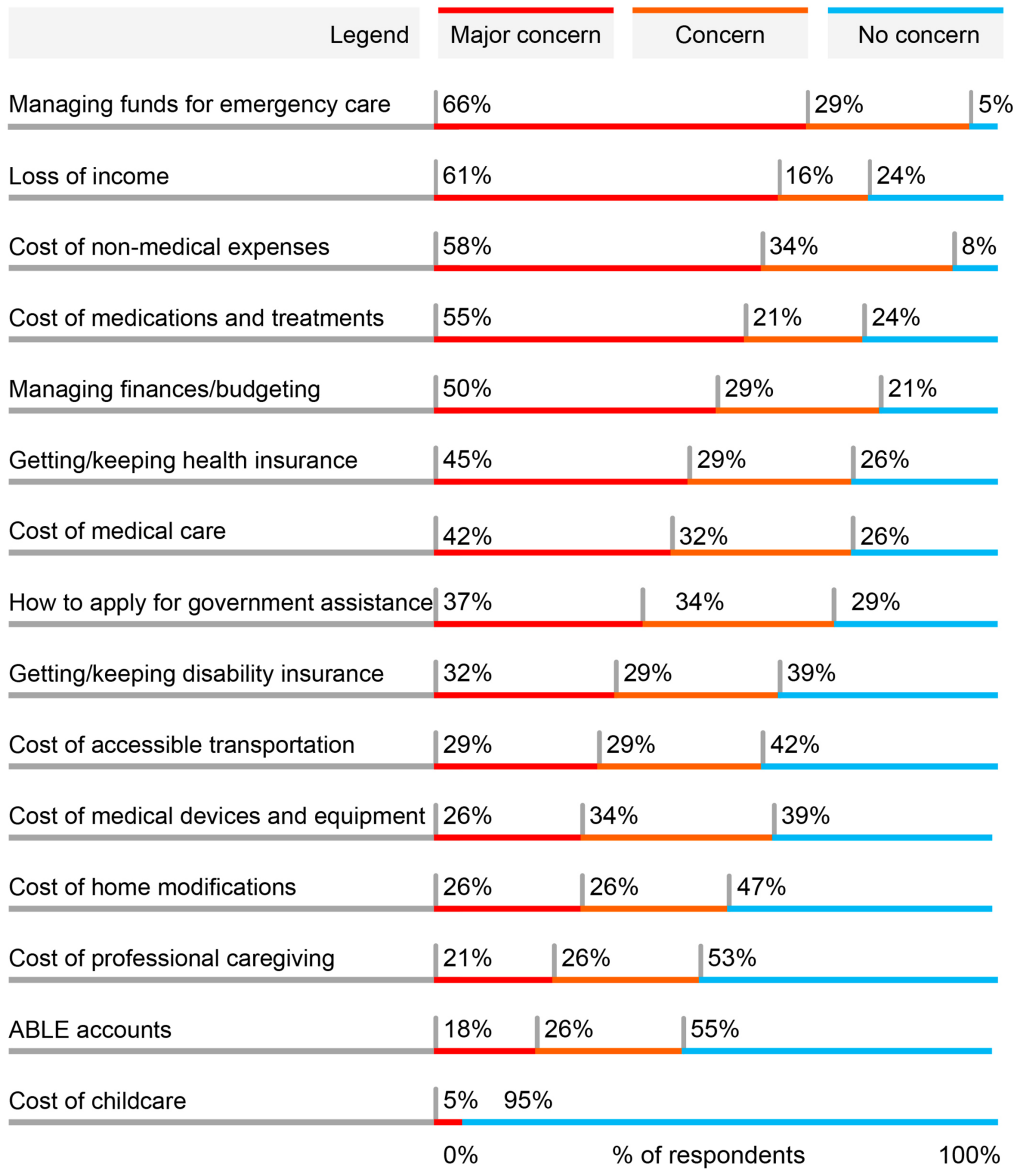
Most common major economic concerns for individuals living with gMG and SDOH barriers. Respondents (*N* = 38) were asked to indicate to what degree each of these economic aspects of living with gMG had been a concern for them since being diagnosed with gMG. Respondents were allowed to choose one of 3 options for each statement: major concern, concern, or no concern. The order of statements was randomized in the survey. Results are shown in order of the proportion reporting the statement as a major concern. Percentages may not total to 100% due to rounding. ABLE, Achieving a Better Life Experience; gMG, generalized myasthenia gravis.

Five types of economic concerns were highlighted as a major concern by at least 50% of the 38 total respondents participating in the quantitative survey (Figure 1). The most common major concern was managing funds for emergency care (66%), with a total of 95% of respondents concerned to any extent (66% [major concern] + 29% [concern]). Cost of medications and treatments, another direct medical cost, was a major concern for 55% of respondents and a concern to any extent for 76%.

Loss of income, an indirect cost, was the second most common major concern, with a total of 77% concerned to any extent. Additionally, the cost of non-medical expenses was a major concern for 58% of patients, with a larger total of 92% of respondents concerned to any extent. A broader concern of managing everyday finances and budgets was a major concern for 50% of respondents, with 79% concerned to any extent. Among other economic concerns, several other direct non-medical expenses were expressed as a major concern by a subgroup of respondents, including the cost of accessible transportation, home modifications, professional caregiving, and childcare.

### 3.4. Sub-analysis of each economic concern by SDOH subgroup

**Figure 2:**
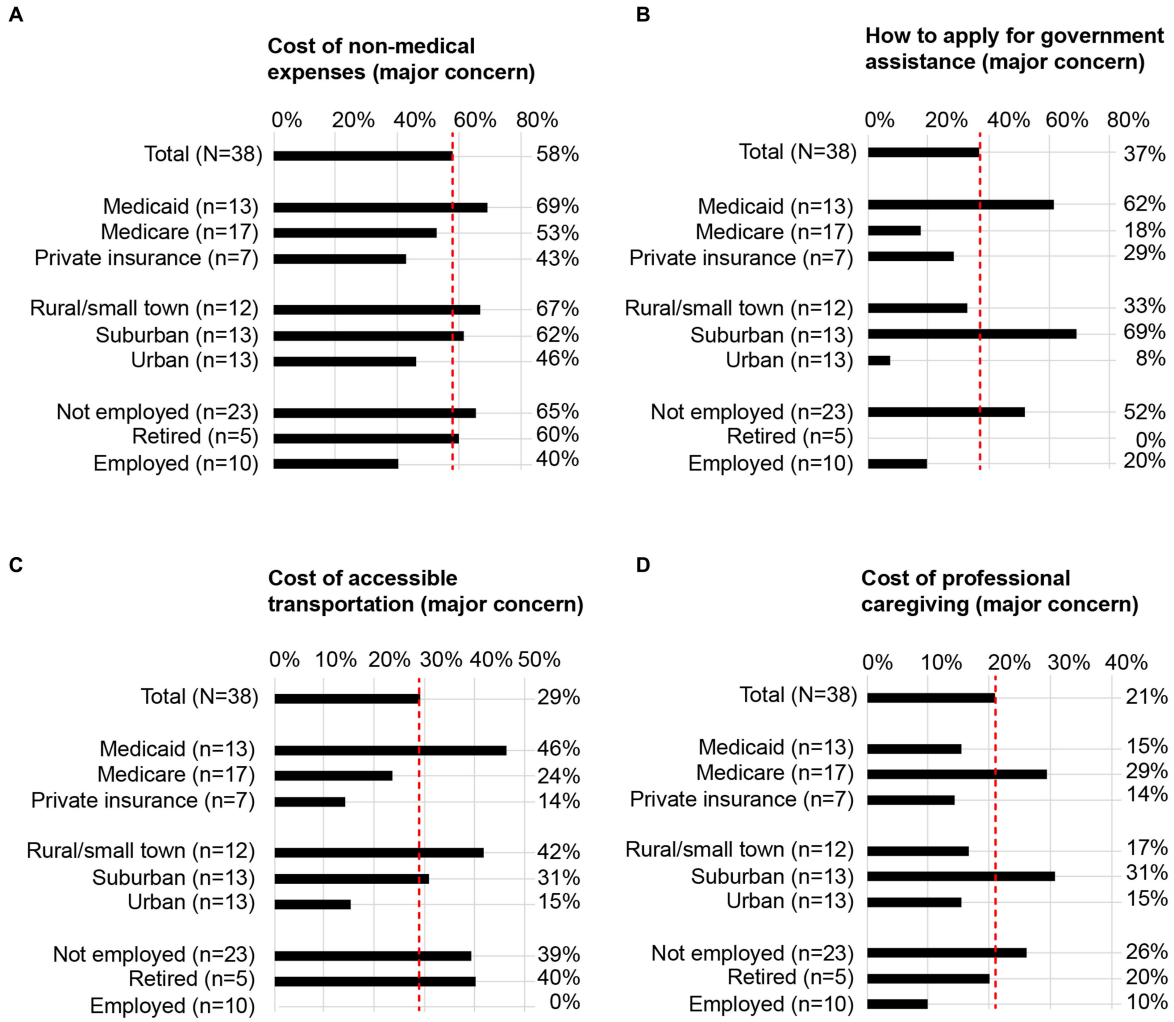
SDOH subgroup analyses for 4 representative economic concerns. For 4 representative statements, the proportion of individuals who self-identified with each pre-specified SDOH subgroup and indicated the statement as a major concern are illustrated in the bar graphs. Data corresponding to the remaining statements are included in Table 2. **(A)** Cost of non-medical expenses as a major concern; **(B)** how to apply for government assistance as a major concern; **(C)** cost of accessible transportation as a major concern; **(D)** cost of professional caregiving as a major concern. Dotted red lines are aligned to the proportion of total respondents (N = 38) who expressed the statement as a major concern. SDOH, social determinants of health.

To gain better insights into potential SDOH drivers of these common economic challenges, the proportions of respondents in each SDOH subgroup who expressed each statement as a major concern were evaluated. Among each pre-specified SDOH subgroup, variations were observed for most statements (Table 2). For instance, managing funds for emergency care was more commonly a major concern among individuals using Medicaid compared with those using private insurance or Medicare. Across 4 of the economic concerns, particularly pronounced differences were observed between SDOH subgroups, where additional barriers were associated with public insurance (Medicare or Medicaid), non-urban living environments (suburban or rural/small town), and non-employment (not employed or retired; Figure 2).

**Table 2:** Sub-analysis of each economic concern by pre-defined SDOH subgroup.

	Total (N = 38)	Insurance type			Living environment			Employment status			Level of education		Race/ethnic background	
		Medicaid (n = 13)	Medicare (n = 17)	Private insurance (n = 7)	Rural/small town (n = 12)	Suburban (n = 13)	Urban (n = 13)	Not employed (n = 23)	Retired (n = 5)	Employed (n = 10)	GED/high school (n = 11)	Post-secondary (n = 23)	Non-White/Caucasian (n = 23)	White/Caucasian (n = 15)
Managing funds for emergency care	66	77	59	71	67	69	62	65	60	70	45	74	65	67
Loss of income	61	77	41	71	58	69	54	70	0	70	45	70	57	67
Cost of non-medical expenses	58	69	53	43	67	62	46	65	60	40	55	61	57	60
Cost of medications and treatments	55	46	59	57	75	62	31	57	60	50	45	52	52	60
Managing finances/budgeting	50	54	41	57	58	62	31	61	0	50	55	48	48	53
Getting/keeping health insurance	45	54	41	43	33	62	38	52	0	50	27	48	52	33
Cost of medical care	42	38	47	43	58	38	31	52	20	30	18	52	43	40
How to apply for government assistance	37	62	18	29	33	69	8	52	0	20	55	26	39	33
Getting/keeping disability insurance	32	38	35	14	33	31	31	39	20	20	18	43	26	40
Cost of accessible transportation	29	46	24	14	42	31	15	39	40	0	36	26	30	27
Cost of home modifications	26	31	29	14	42	8	31	30	20	20	18	35	22	33
Cost of medical devices and equipment	26	23	35	14	25	31	23	30	20	20	18	26	22	33
Cost of professional caregiving	21	15	29	14	17	31	15	26	20	10	18	22	26	13
ABLE accounts	18	31	18	0	17	23	15	22	20	10	18	17	26	7
Cost of childcare	5	8	6	0	8	0	8	9	0	0	0	9	9	0

Proportion of respondents in each SDOH subgroup reporting the statement as a major concern, %. ABLE, Achieving a Better Life Experience; GED, General Educational Development; SDOH, social determinants of health.

#### 3.4.1. Cost of non-medical expenses

Individuals using Medicaid, living in non-urban living environments, and not employed or retired more commonly expressed the cost of non-medical expenses as a major concern compared with other SDOH subgroups (Figure 2A; Table 2).

#### 3.4.2. Applying for government assistance

Individuals using Medicaid, living in a suburban living environment, and not employed more commonly expressed applying for government assistance as a major concern compared with other SDOH subgroups (Figure 2B; Table 2).

#### 3.4.3. Cost of accessible transportation

Individuals using Medicaid, living in rural/small town living environments, and not employed or retired more commonly expressed the cost of accessible transportation as a major concern compared with other SDOH subgroups (Figure 2C; Table 2).

#### 3.4.4. Cost of professional caregiving

Individuals using Medicare, living in a suburban living environment, and not employed more commonly expressed the cost of professional caregiving as a major concern compared with other SDOH subgroups (Figure 2D; Table 2).

## 4. Discussion

In the present study, we sought to better understand the compounding economic burden of living with gMG and SDOH barriers by gaining firsthand patient perspectives from a diverse group of individuals living with gMG in the US. While previous studies have substantially increased our understanding of the economic burden of gMG across the globe, to our knowledge, this study is the first to capture the voices of historically underrepresented individuals with gMG who are living with additional SDOH challenges in the US. Better characterizing the nuanced experiences of this subgroup is crucial to fill current evidence gaps, which have arisen in part due to systematic exclusion of socially disadvantaged individuals from traditional research approaches. For instance, studies using pharmaceutical claims databases may exclude individuals who are uninsured or undertreated, and studies based on large academic institutions may be selecting for individuals who have access to such sites of care. By using unique and targeted recruiting approaches to capture and highlight the experiences of such individuals, we begin to address an important evidence gap to fuel future steps for the gMG care community to ensure better support and access to healthcare for all individuals affected.

### 4.1. The overall economic burden of individuals living with gMG and SDOH challenges

Our results demonstrated a high degree of concern for both direct (medical and non-medical) and indirect costs among individuals living with gMG and SDOH barriers. The impact of direct costs is not surprising given the high costs associated with gMG globally (11, 14–16, 21, 23, 24, 28, 43). In particular, it was expected that the cost of emergency care would be the most common major economic concern, given the significant increase in costs and healthcare resource utilization associated with myasthenic crises and exacerbation events reported in the US (24). In clinical practice, these serious events are observed more commonly early in the disease course after diagnosis and are often associated with poorly controlled disease. While traditional gMG treatment approaches that focus on achieving good long-term outcomes are important, the potential additional benefits of diagnosing and gaining control of the disease as rapidly as possible should be further studied, as it may help improve short-term outcomes, reduce hospitalizations and associated costs, and alleviate burden of individuals living with gMG.

Notably, loss of income, an indirect cost, was a major driver of economic concerns, with individuals who were not employed/retired reporting pronounced challenges across multiple economic concerns. This is consistent with previous reports associating high burden with loss of employment in individuals living with gMG (10, 44, 45). In a study based in Germany, over 70% of 1,660 survey participants experienced limitations regarding employment due to gMG, with reasons including incapacity to work or recurrent occupational and/or professional disability (10). In Japan, 27.2% of 917 individuals living with gMG experienced unemployment, while 35.9% experienced a decrease in income (46). Similar associations between gMG and employment challenges have been observed in Italy (47), Denmark (32), and Australia (33), with a recent meta-analysis reporting that on a global scale, 50% of individuals living with gMG may be not employed (48). Taken together with our results, further characterizing the impact of indirect costs should be prioritized in gMG. As loss of income may be causal to or exacerbate other economic concerns, it is also imperative to address alternative employment options and solutions for individuals living with gMG, including those living with SDOH barriers.

### 4.2. Individuals living with gMG who are not employed, using public insurance, and/or living in non-urban environments may face pronounced economic challenges

In addition to individuals who were not employed, pronounced challenges were also observed for individuals using public insurance (Medicaid or Medicare) or living in non-urban environments (suburban or rural/small town). The types of economic concerns that particularly highlighted these differences included direct non-medical costs and applying for government assistance, which was a unique finding given that much of the literature around the economic burden of gMG focuses on direct medical costs associated with treatments and healthcare utilization.

While pronounced economic burden in individuals living with gMG who are not employed can be expected from existing evidence, the compounding challenges associated with certain insurance types and living environments have been much less characterized in gMG. Low income could in part explain pronounced challenges for Medicaid users, while increased comorbidities could contribute to exacerbation of disability-related economic concerns for Medicare users (49, 50). Individuals living in non-urban environments may have poorer access to or longer distances to travel to gMG specialists, treatment centers, institutions to apply for support/assistance programs, and patient communities. To confirm these hypotheses, further research is necessary.

Although the impact of non-medical expenses on economic burden can often be overshadowed by substantial direct medical costs, our study highlights the major day-to-day burden that non-medical costs impose on individuals living with gMG and SDOH barriers. Certain SDOH can act as subtle or “silent” barriers to accessing non-medical resources that are frequently assumed to be utilized by the majority, such as government assistance (51). Proactively addressing such barriers could help begin providing solutions for the wide range of economic concerns we observed in our study, which can potentially also alleviate negative mental health effects (6) and caregiver burden associated with gMG (11, 21).

### 4.3. Clinician perspectives and call to action

While the present analysis could guide gMG clinicians to identify specific SDOH risks associated with pronounced economic challenges, many may already be aware of these issues through their day-to-day practice. In fact, in a survey conducted among >1,500 US physicians, financial instability and transportation problems were the top 2 SDOH challenges physicians believed their patients experienced (52). The larger challenge appears to be finding adequate and relevant assistance to address them, as clinicians have limited time and resources to directly support diagnosed individuals beyond the clinical realm. In the same survey, >60% of physicians reported they have little to no time and ability to effectively address SDOH challenges, with >75% expressing inadequateness or lack of accessible community resources and the workforce to direct patients to them as major obstacles (52). Strikingly, only 19% of respondents reported that they had adequate resources in their practice to address SDOH challenges (52). To address these issues, increased involvement of case managers or social workers who not only support hospital discharges and prevention of rehospitalizations but also are knowledgeable about broad and local assistance programs such as transportation, social services, and food banks have been increasingly prioritized in chronic illnesses (53–56). Many of these programs, including Chronic Care Management, have been demonstrated to improve health outcomes while potentially saving costs (53–56). Indeed, better outcomes, including reduced hospital-level resource utilization and costs, have been observed with holistic care management interventions in multiple sclerosis (MS) (57–59) and stroke (60–63). Robust resourcing of case managers and social workers could also allow providers to focus their time on clinical care, further benefiting the healthcare system. However, currently such support is often insufficient or deprioritized for gMG, even at some of the largest healthcare institutions.

Many clinicians have been, and are currently, advocating to improve such on-site support resources for gMG. In addition to financial resources, psychosocial and mental health support is a priority, as individuals diagnosed with gMG face the burden of a lifelong chronic disease, often causing emotional strain, anxiety, and depression that can foster additional barriers to appropriate care, disease management, and survival (47, 64). While little is currently offered at gMG diagnosis at most institutions, long-term psychosocial support, analogous to that offered with cancer diagnoses, can improve outcomes for gMG via an increasingly personalized approach (47, 64). To further accelerate such holistic support in gMG on a broader scale, a large collaborative effort in gMG, modeling historical success and ongoing initiatives in other therapy areas, is needed. First, individuals living with gMG and PAGs should be further empowered to advocate for their needs through increased disease education and closer partnerships (65–67). The active engagement of individuals diagnosed with gMG in their own clinical decision-making processes is highly valuable, and an integral component of the patient-centered care model. To further improve outcomes with personalized care solutions in gMG, diagnosed individuals should be provided the right information, tools, and support to discuss their priorities and concerns with their clinicians, collaborate with appropriate support teams, and feel confident in their treatment and management plans. In addition, settings in which individuals living with gMG, caregivers, clinicians, nurses, PAGs, decisionmakers, and other stakeholders in gMG are able to discuss real-world issues and connect with each other should be implemented in gMG. Although social media networks can be useful for this, amplified voices can often be biased toward strongly negative or positive opinions, missing a large part of the community who may not be active participants. More formal opportunities including regular, large-scale conferences can be highly valuable in identifying actionable needs, leading to successful consensus development as in the case of MS (68, 69). While the Myasthenia Gravis Foundation of America and several similar organizations provide excellent groundwork, the broader gMG community must improve cross-organizational collaborations globally to strengthen the collective voice that has been historically fragmented across the world.

To ultimately improve outcomes for an increasingly diverse population of patients with gMG, current unmet needs must be clearly communicated to decision-makers who influence systematic and institutional resource allocations. In addition to researchers generating critical evidence, a unified voice from the gMG community will be the key to instigating broader-scale changes in gMG support. Such grassroots movements have been successful in other therapy areas such as the MS Brain Health initiative, where structured discussions of a global author group comprising clinicians, researchers, specialist nurses, health economists, and patient group representatives culminated in evidence-based policy recommendations endorsed by over 40 professional MS associations and PAGs (70). While collectively empowering the gMG community to a similar extent may require time and innovative collaboration strategies, accelerating such proactive actions in the gMG community will be a critical next step to significantly impact outcomes for individuals living with gMG sooner, and more comprehensively, than ever before.

### 4.4. Limitations

Several limitations to our study should be noted. Although our study sample comprised one of the most diverse groups of individuals living with gMG in the US, the cohort size limited the scope of the analysis. For example, while differences observed between subgroups based on level of education and race/ethnicity were unremarkable in the present study, an expanded sample size could uncover additional insights. As the cohort size also limited statistical analyses, results presented in this report reflect descriptive trends, with larger data sets required to generalize any conclusions. Secondly, the screener included questions that were used to classify respondents into pre-defined SDOH subgroups and were not comprehensive. As a result, our findings should not be interpreted to be representative of that of a larger, better characterized population (e.g., while cost of childcare was assessed as a potential concern, whether the respondent was living with a child with childcare needs was unknown). Lastly, time since diagnosis was not captured in the survey to avoid confounding results due to recall bias in self-reporting. Thus, further studies are required to assess the impact of disease characteristics (including duration of disease, severity, etc.) on economic burden among individuals living with gMG and SDOH challenges.

## 5. Conclusion

Although our understanding of the economic burden of individuals living with gMG both globally and in the US is drastically improving, evidence based on traditional research methods systematically excluded the voices of key subgroups that may be facing disproportionate economic challenges and unique unmet needs. The present study aimed to address this evidence gap to better understand the impact of SDOH challenges in gMG from the patient’s perspective, by using unique approaches to recruit a highly diverse cohort of individuals living with gMG. Individuals living with gMG and SDOH challenges commonly demonstrated high levels of concern for not only direct medical costs, as expected, but also indirect costs and direct non-medical costs across multiple dimensions of their everyday lives. Additionally, pronounced economic challenges were observed in individuals who were not employed, using public insurance, and/or living in non-urban environments. These results highlight that additional and targeted support should be provided for individuals living with gMG and certain SDOH challenges. However, currently offered access and support resources may inadequately address many of the multifaceted issues that can impede better health outcomes for individuals with gMG. While further research is required to confirm and extend our findings, holistic improvements in institution- and policy-level healthcare structures and processes may be crucial to improve support, access, and outcomes for not only individuals living with gMG impacted by SDOH challenges but also the larger gMG community.

## Data availability statement

The original contributions presented in the study are included in the article/Supplementary material, further inquiries can be directed to the corresponding author.

## Ethics statement

The studies involving humans were approved by WCG IRB, Puyallup, Washington, USA. The studies were conducted in accordance with the local legislation and institutional requirements. The participants provided their written informed consent to participate in this study.

## Author contributions

TH, SS, and GP were involved in developing the concept and designing the study methodology. MS and SS oversaw data collection and analysis. All authors were involved in interpretation of the results. JH, NS, AA, and JG provided critical clinician perspectives. MS drafted the manuscript. All authors contributed to the article and approved the submitted version.

## Funding

This study was funded by Argenx US Inc. (Boston, MA, USA). The funder was involved in the study design, interpretation of aggregate results, manuscript reviews, and decision to submit for publication.

## Conflict of interest

TH, JG, and GP are employees of Argenx (Boston, MA, USA). JH has received research support (paid to his institution) from Alexion Pharmaceuticals, Argenx, Cartesian Therapeutics, the Centers for Disease Control and Prevention (Atlanta, GA, USA), the Myasthenia Gravis Foundation of America, the Muscular Dystrophy Association, the National Institutes of Health (including the National Institute of Neurological Disorders and Stroke and the National Institute of Arthritis and Musculoskeletal and Skin Diseases), PCORI, Ra Pharmaceuticals (now UCB Biosciences), and Takeda Pharmaceuticals; and honoraria from Alexion Pharmaceuticals, argenx, F. Hoffman-LaRoche Ltd., Immunovant Inc., Merck EMD Serono, NMD Pharma, Novartis Pharmaceuticals, Ra Pharmaceuticals (now UCB Biosciences), Regeneron Pharmaceuticals, and Sanofi US; and non-financial support from Alexion Pharmaceuticals, argenx, Ra Pharmaceuticals (now UCB Biosciences) and Toleranzia AB. NS consults for argenx, Alexion Pharmaceuticals, UCB Biosciences, and Immunovant Inc. and is a speaker for argenx, Alexion Pharmaceuticals, and UCB Biosciences. AA is a Patient Education Speaker for argenx. MS and SS are employees of ZS Associates (New York, NY, USA) and serve as paid consultants for argenx.

## Publisher’s note

All claims expressed in this article are solely those of the authors and do not necessarily represent those of their affiliated organizations, or those of the publisher, the editors and the reviewers. Any product that may be evaluated in this article, or claim that may be made by its manufacturer, is not guaranteed or endorsed by the publisher.
