# Supplementary material for: The economic burden of individuals living with generalized myasthenia gravis and facing social determinants of health challenges
Source: Front Public Health. 2023 Sep 12;11:1247931. doi: 10.3389/fpubh.2023.1247931 (PMC10520715; doi:10.3389/fpubh.2023.1247931)
Supplement: Supplementary file 1 [file Table_1.DOCX]

Supplementary Material

The Economic Burden of Individuals Living with Generalized Myasthenia Gravis and Facing Social Determinants of Health Challenges

Tom Hughes^*^, James F. Howard Jr., Nicholas J. Silvestri, Ashley E.L. Anderson, Mai Sato, Sharon Suchotliff, Jeffrey T. Guptill, Glenn Phillips

***Correspondence:** Tom Hughes [thughes@argenx.com](mailto:thughes@argenx.com)

[1 Supplementary Table 1. Inclusion criteria and screening parameters for individuals living with gMG. 2](#_Toc139722818)

[2 Supplementary Table 2. Inclusion criteria for caregivers and PAG representatives recruited for qualitative interviews. 3](#_Toc139722819)

[3 Supplementary Table 3. Interview discussion guides. 3](#_Toc139722820)

[4 Supplementary Table 4. Quantitative survey content.* 4](#_Toc139722821)

# Supplementary Table 1. Inclusion criteria and screening parameters for individuals living with gMG.

| **Inclusion criteria** | **Custom screening parameters to ensure diverse sample** |
| --- | --- |
| Currently living with gMG | All |
| Have been or currently on treatment to manage gMG | All |
| Currently reside in US state or territory | All |
| Aged 18-75 years | Screened to include a distribution of patients aged 18-39, 40-59, and 60-75 years of age |
| Able to disclose gender | Screened to include a balanced distribution of gender identities |
| Able to disclose employment status | Screened to include a distribution of employed and unemployed individuals |
| Able to disclose ethnic and racial background | Screened to include a distribution of patients identifying as White or Caucasian, Hispanic or Latin@, African American or Black, Native American or Indigenous Person, Asian or Pacific Islander, and Middle Eastern or North African |
| Able to disclose living environment | Screened to include a distribution of patients identifying as living in an urban, suburban, or rural environment |
| Able to disclose level of education | Screened to include a distribution of patients with level of education of high school/GED or post-secondary education |
| Able to disclose income, and annual family income is ≤$100,000 | For participants identifying as White or Caucasian, annual family income was capped at ≤$75,000 for inclusion in the study |

GED, General Educational Development; gMG, generalized myasthenia gravis; US, United States.

# Supplementary Table 2. Inclusion criteria for caregivers and PAG representatives recruited for qualitative interviews.

| **Inclusion criteria for caregivers of patients living with gMG (n=2)** | **Inclusion criteria for gMG PAG representatives (n=2)** |
| --- | --- |
| - Currently caring for someone living with gMG aged 18-75 years - Patient under care has used at least one treatment for gMG - Is a family, relative, friend, or professional caregiver of a patient living with gMG - Gives regular care or ad hoc support for a patient with gMG - Last known education status of patient being cared for is high school, GED, some college, or associate degree - Annual household income of patient being cared for is ≤$100,000 (or ≤$75,000 if patient is White or Caucasian) - Able to disclose gender and employment status of the patient cared for | - Resides in a US state or territory - Had served or is serving as a member of a diverse community of patients with gMG, including members of any or all diverse racial and ethnic backgrounds - Has been working with or for a gMG PAG for ≥3 years - Personally interacted with ≥5 patients receiving assistance or services for gMG in the last 60 days |

GED, General Educational Development; gMG, generalized myasthenia gravis; PAG, patient advocacy group; US, United States.

# Supplementary Table 3. Interview discussion guides.

| **Individuals living with gMG and caregivers** | **PAG representatives** |
| --- | --- |
| - Introduction and background (5 minutes)   - Market research permissions, consent, and disclosures   - Research objectives   - Review of pre-interview exercise sheet - Experience and challenges at diagnosis and treatment decision-making stage (12 minutes) - Experience and challenges post-diagnosis (12 minutes) - Experience and challenges with existing patient support (12 minutes) - Closing (4 minutes) | - Introduction and background (5 minutes)   - Market research permissions, consent, and disclosures   - Research objectives - Unique needs of patients with gMG and their caregivers (10 minutes) - Advocacy activities and approaches to patient and caregiver support in gMG (12 minutes) - Current gMG support landscape and key takeaways (15 minutes) - Closing (2 minutes) |

gMG, generalized myasthenia gravis; PAG, patient advocacy group.

# Supplementary Table 4. Quantitative survey content.*

| **Section 1: General gMG experiences** | **Section 2: Experiences at 4 different timepoints through their diagnosis and treatment** |
| --- | --- |
| - Day-to-day challenges and impact on physical, mental, and emotional wellbeing - Perception of experience managing gMG and engaging with their healthcare providers - Frequently used sources of gMG-related information and perception of support | - Specific challenges, resources used, and resources needed at diagnosis - Specific challenges, resources used, and resources needed when accessing treatment - Specific challenges, resources used, and resources needed when initiating treatment - Specific challenges, resources used, and resources needed in continuing treatment and life with gMG |

*Additional results from the survey not presented in this report have been previously published (Hughes T, Anderson AEL, Habib AA, Perez K, Bergin C, Suchotliff S, et al. Impact of social determinants of health on individuals living with generalized myasthenia gravis and implications for patient support programs. *Front Public Health.* (2023) 11:1147489. doi: 10.3389/fpubh.2023.1147489).

gMG, generalized myasthenia gravis.
